# Benefits of Adjuvant Chemotherapy Differ by Menopausal Status in Women with HR+/HER2- Early Breast Cancer, 1-3 Positive Nodes, and a Low Recurrence Score

**DOI:** 10.1093/oncolo/oyac012

**Published:** 2022-03-28

**Authors:** Anne Jacobson

## Abstract

Adjuvant chemotherapy is beneficial for premenopausal women-and not postmenopausal women-with HR-positive, HER2-negative, early breast cancer with 1-3 lymph nodes and a recurrence score of 0-25, according to updated results from the phase III RxPonder study.

Multigene prognostic scores hold the potential to guide treatment decisions, including the decision to forgo certain therapy when the risks outweigh the benefits. The 21-gene Oncotype DX recurrence score has been used to identify which women with node-negative, hormone receptor (HR)-positive, human epidermal growth factor receptor 2 (HER2)-negative early breast cancer can safely forgo chemotherapy without increasing the risk of recurrence.

The phase III Southwest Oncology Group (SWOG) S1007 RxPonder trial was designed to evaluate the benefit of chemotherapy added to standard adjuvant endocrine therapy in women with node-positive, HR-positive, HER2-negative early breast cancer with an Oncotype DX® recurrence score of 0-25. Preliminary findings from the RxPonder trial suggested that postmenopausal women can avoid adjuvant chemotherapy without compromising long-term clinical outcomes.^1^

In the current analysis, Kevin Kalinsky, M.D., of Emory University, presented updated results from the ongoing RxPonder trial.^2,3^

## Study Design

The SWOG S1077 RxPonder trial enrolled 5,018 patients with HR-positive, HER2-negative breast cancer with 1-3 positive axillary lymph nodes (nodal stage N1) and no distant metastasis. All patients had an Oncotype DX recurrence score of 0-25, indicating a low risk of recurrence. Patients were randomly assigned to treatment with chemotherapy plus standard adjuvant endocrine therapy (*n* = 2,507) or endocrine therapy alone (*n* = 2,511). The primary endpoint was invasive disease-free survival (IDFS).

The median follow-up in the current analysis was 5.3 years. Baseline characteristics were similar in both treatment groups. The median patient age was 57.5 years (range, 18.3 to 87.6 years). At enrollment, one-third of patients (33.2%) were premenopausal and two-thirds (66.8%) were postmenopausal. Most patients (65.3%) had 1 positive node; 25.2% had 2 positive lymph nodes, and 9.2% had 3 positive nodes.

## Key Findings

Results showed a statistically significant interaction between chemotherapy benefit, as measured by IDFS, and menopausal status (*p* =.008) (Table 1). Among postmenopausal women, the addition of chemotherapy did not improve IDFS at 5 years compared with endocrine therapy alone (91.3% vs. 91.9%). The hazard ratio (HR) for invasive disease recurrence, new primary breast or other cancer, or death was 1.02 (*p* =.89).

**Table 1.** Invasive disease-free survival by adjuvant therapy regimen and menopausal status

Patient subgroup	Chemotherapyplus ET (*n* = 2,487)	ET alone (*n* = 2,497)	HR (95% CI)	*p* value
All patients	92.2%	91.0%	0.86 (0.72-1.03)	.10
Postmenopausal	91.3%	91.9%	1.02 (0.82-1.26)	.89
Premenopausal	93.9%	89.0%	0.60 (0.43-0.83)	.002

Abbreviations: CI, confidence interval; ET, endocrine therapy; HR, hazard ratio; IDFS, invasive disease-free survival.

In contrast, chemotherapy was associated with a significant improvement in IDFS among premenopausal women. In this subgroup, the 5-year IDFS was 93.9% among those who received chemotherapy plus endocrine therapy and 89.0% for those who received endocrine therapy alone. This represents a 40% reduction in the risk of IDFS events with the addition of chemotherapy to adjuvant endocrine therapy (HR, 0.60; *p* =.002).

Distant relapse-free survival (DRFS) outcomes followed a similar pattern (Table 2). Among postmenopausal women, 5-year DRFS was 94.4% in both treatment groups (HR, 1.05; *p* =.70). By comparison, premenopausal women experienced a 42% reduction in the risk of DRFS events with the addition of chemotherapy to endocrine therapy. The 5-year DRFS rate was 96.1% in the chemotherapy plus endocrine therapy group and 92.8% for those treated with endocrine therapy alone (HR, 0.58; *p* =.009).

**Table 2.** Distant relapse-free survival by adjuvant therapy regimen and menopausal status

Patient subgroup	Chemotherapy plus ET (*n* = 2,487)	ET alone (*n* = 2,497)	HR (95% CI)	*p* value
All patients	94.9%	93.9%	0.88 (0.71-1.09)	.25
Postmenopausal	94.4%	94.4%	1.05 (0.81-1.37)	.70
Premenopausal	96.1%	92.8%	0.58 (0.39-0.87)	.009

Abbreviations: CI, confidence interval; DRFS, disease relapse-free survival; ET, endocrine therapy; HR, hazard ratio.

Among premenopausal women, the benefit of chemotherapy was consistent across all patient subgroups, regardless of age, disease grade, tumor size, node status, or recurrence score. The relative benefit of chemotherapy did not increase as recurrence scores increased from 0 to 25.

According to Dr. Kalinsky and colleagues, the updated and peer-reviewed findings from the RxPonder trial suggest that postmenopausal women HR-positive, HER2-negative early breast cancer with 1-3 positive lymph nodes and an Oncotype DX recurrence score of 0-25 can safely forgo adjuvant chemotherapy without compromising long-term IDFS and DRFS. Moreover, premenopausal women with the same characteristics are likely to benefit from chemotherapy added to adjuvant endocrine therapy.

## Notes

Please note that this content was created for the purpose of summarizing the content of the San Antonio Breast Cancer Symposium 2021 and has not been peer reviewed under the standard policies of *The Oncologist*.

## References

[1] Kalinsky K , Barlow WE, Meric-Bernstam F, et al. SWOG S1007: Adjuvant trial randomized ER+ patients who had a recurrence score <25 and 1-3 positive nodes to endocrine therapy (ET) versus ET + chemotherapy. Presented at the 2020 San Antonio Breast Cancer Symposium (SABCS). December 8-11, 2020. Abstract GS3-01.

[2] Kalinsky KM , Barlow WE, Gralow JR, et al. Updated results from a phase 3 randomized clinical trial in participants (pts) with 1-3 positive lymph nodes (LN), hormone receptor-positive (HR+) and HER2-negative (HER2–) breast cancer (BC) with recurrence score (RS) <25 randomized to endocrine therapy (ET) +/– chemotherapy (CT): SWOG S1007 (RxPONDER). Presented at the 2021 San Antonio Breast Cancer Symposium (SABCS). December 7-10, 2021. Abstract GS2-07.

[3] Kalinsky K , Barlow WE, Gralow JR, et al. 21-Gene assay to inform chemotherapy benefit in node-positive breast cancer. N Engl J Med. 2021;385:2336-2347. doi: 10.1056/NEJMoa2108873. 34914339 10.1056/NEJMoa2108873 PMC9096864

